# Fine-scale vertical relationships between environmental conditions and sound scattering layers in the Southwestern Tropical Atlantic

**DOI:** 10.1371/journal.pone.0284953

**Published:** 2023-08-04

**Authors:** Ramilla Assunção, Anne Lebourges-Dhaussy, Alex C. da Silva, Gildas Roudaut, Alejandro Ariza, Leandro N. Eduardo, Syumara Queiroz, Arnaud Bertrand

**Affiliations:** 1 Laboratório de Oceanografia Física Estuarina e Costeira, Departamento de Oceanografia, UFPE, Recife, Pernambuco, Brazil; 2 LEMAR, UBO, IFREMER, IRD, CNRS, Technopole Brest Iroise, Plouzané, France; 3 MARBEC, Université Montpellier, CNRS, Ifremer, IRD, Sète, France; 4 DECOD (Ecosystem Dynamics and Sustainability), IFREMER, INRAe, Institut-Agro—Agrocampus Ouest, Nantes, France; 5 Universidade Federal Rural de Pernambuco, Recife, Pernambuco, Brazil; The University of Auckland - City Campus: University of Auckland, NEW ZEALAND

## Abstract

Ocean dynamics initiate the structure of nutrient income driving primary producers, and these, in turn, shape the distribution of subsequent trophic levels until the whole pelagic community reflects the physicochemical structure of the ocean. Despite the importance of bottom-up structuring in pelagic ecosystems, fine-scale studies of biophysical interactions along depth are scarce and challenging. To improve our understanding of such relationships, we analyzed the vertical structure of key oceanographic variables along with the distribution of acoustic biomass from multi-frequency acoustic data (38, 70, and 120 kHz) as a reference for pelagic fauna. In addition, we took advantage of species distribution databases collected at the same time to provide further interpretation. The study was performed in the Southwestern Tropical Atlantic of northeast Brazil in spring 2015 and autumn 2017, periods representative of canonical spring and autumn conditions in terms of thermohaline structure and current dynamics. We show that chlorophyll-*a*, oxygen, current, and stratification are important drivers for the distribution of sound scattering biota but that their relative importance depends on the area, the depth range, and the diel cycle. Prominent sound scattering layers (SSLs) in the epipelagic layer were associated with strong stratification and subsurface chlorophyll-*a* maximum. In areas where chlorophyll-*a* maxima were deeper than the peak of stratifications, SSLs were more correlated with stratification than subsurface chlorophyll maxima. Dissolved oxygen seems to be a driver in locations where lower oxygen concentration occurs in the subsurface. Finally, our results suggest that organisms seem to avoid strong currents core. However, future works are needed to better understand the role of currents on the vertical distribution of organisms.

## 1. Introduction

Acoustic echosounders are widely used to detect a variety of environmental and biological components in pelagic ecosystems, from oceanic microstructure to mesoscale eddies, and from zooplankton to top predators [1–4]. Sound scattering layers (SSLs) are vertically discrete water column aggregations of organisms commonly detected during acoustic surveys [5, 6]. Most of the signals within SSLs come from resonant gas-bearing species such as swimbladdered fish or siphonophores, and to a lesser extent, from zooplankton with fluid-like acoustic characteristics (e.g., euphausiids, copepods, salps) [1, 7–9].

SSLs vary over a broad range of spatial and temporal scales in response to biological and environmental forcing [10–12]. Beyond large biogeographical constraints, the pelagic ecosystem structure and associated SSLs can quickly respond to physicochemical changes (spatial and/or seasonal) in the water column [2, 13–15]. Ocean forcing initiates the structuring of primary producers and these, in turn, shape the distribution of subsequent trophic levels until the whole pelagic community reflects the physicochemical structure of the ocean [14, 16]. In agreement with this bottom-up structuring, the thermohaline features (e.g., pycnocline, mixed-layer depth—MLD, thermocline thickness, presence of barrier layer) modulate the income of nutrients into the photic layer, controlling primary productivity and thus the vertical and horizontal distribution of organisms [2, 17, 18].

Diel vertical migration (DVM) is another key factor that has long been recognized for driving changes in the SSLs structures [19–21]. Fundamentally, DVM arises from the organisms need to feed in shallow productive waters at night and hide from visual predators at depth when the sun rises [22]. Consequently, vertical migrants are constrained by light penetration from the surface and dissolved oxygen concentrations, temperature, and stratification processes, that determine the habitability and food supply in the water column [1, 10, 12, 23–25]. DVMs are also influenced by biological (endogenous cycle and species composition) factors [1, 12, 24, 25].

Despite the importance of bottom-up processes in the structuring and functioning of pelagic ecosystems, fine-scale studies of biophysical interactions along depth are scarce and challenging. Nets usually do not provide the vertical resolution required to understand fine-scale processes [9], and acoustic studies have been so far mostly restricted to single-frequency interactions with few environmental variables [11, 26]. Therefore, the description, quantification, and understanding of the fine-scale relationships between physical-chemical factors and the distribution of the acoustic biota signal is still an exploratory science. Comprehensive, fine-scale, and multivariate environmental analyses might help understand group-specific biophysical interactions and structure at the scale of meters. In this context, the goal of the present study is to provide the underlying physical context for aggregations of organisms, using acoustic response as a proxy. We discuss why these physical factors are potential drivers for these aggregations.

**Fig 1:**
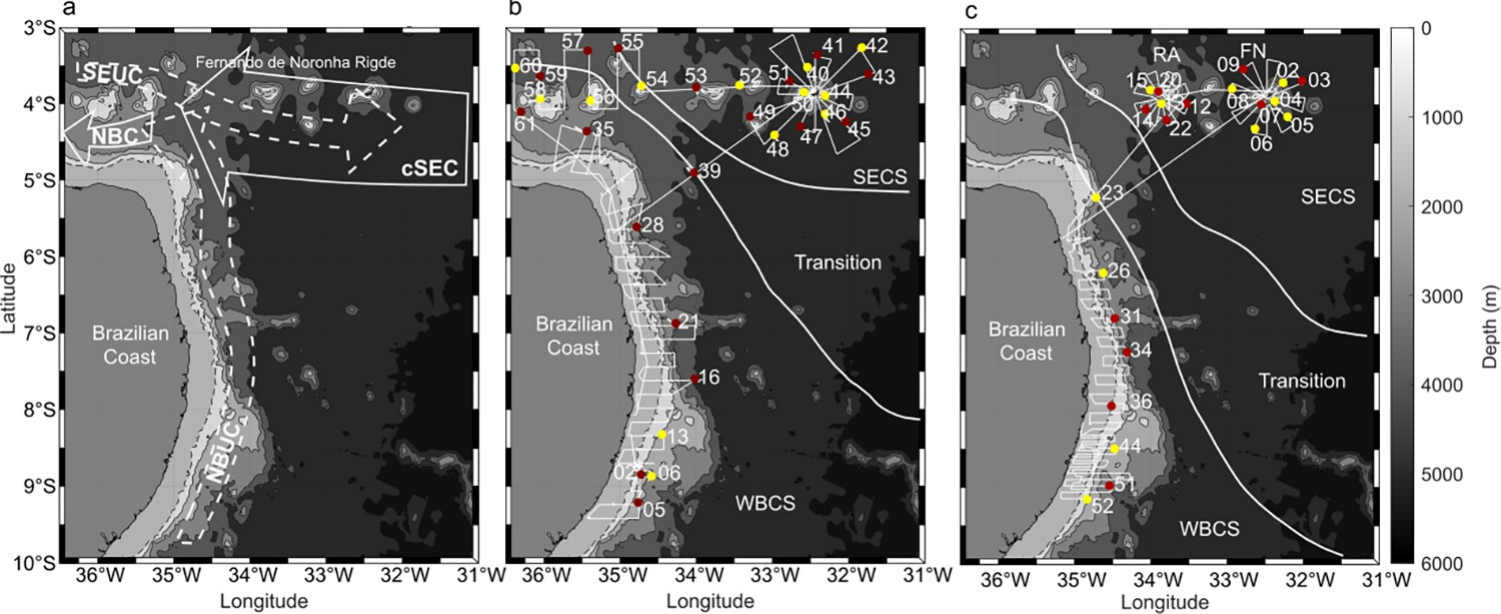
The main currents (a) of each thermohaline system are North Brazil Undercurrent (NBUC), North Brazil Current (NBC), the central branch of South Equatorial current (cSEC), and south equatorial undercurrent (SEUC) [28, 29]. The dotted arrows refer to the subsurface currents and the continuous arrows to the surface currents. Survey tracks (white lines) of ABRACOS 1 (b) and ABRACOS 2 (c) surveys. Dots represent the day (yellow) and night (red) CTD stations used in this study. The continental shelf is represented in light grey; the dashed black line represents the shelf break (70 m isobath); other bathymetric contours (solid black lines) are by 1000 m intervals. RA: Rocas Atoll; FN: Fernando de Noronha archipelago. The boundaries (solid white lines) between the western boundary current system (WBCS), the south equatorial current system (SECS), and the transitional area are plotted according to Assunção et al. (2020).

For that purpose, we take advantage of data collected during two multidisciplinary surveys conducted in the Southwestern Tropical Atlantic (SWTA) in the spring of 2015 and autumn of 2017 (Fig 1). These periods are representative of canonical spring and autumn conditions in terms of thermohaline structure and currents dynamics [27, 28]. This region encompasses different hydrodynamic systems [27–29]. The area along the continental slope (Fig 1) corresponds to a western boundary current system (WBCS) constituted by the North Brazil Undercurrent (NBUC) and the North Brazil Current (NBC). This WBCS is characterised by a thick thermocline and a high barrier layer frequency (BL) [27]. Conversely, the area located along the Fernando de Noronha chain corresponds to the south equatorial current system (SECS) formed by the central branch of South Equatorial current (cSEC) and the south equatorial undercurrent (SEUC). The SECS presents a narrow but well-marked thermocline and low BL frequency [27, 29]. This zoning, which also displays significant seasonal variations, implies the formation of habitats with distinct thermal, saline, hydrodynamic conditions, hosting, in some cases, assemblages of different species [27, 28, 30].

In this context, we investigate fine-scale vertical relationships among fundamental oceanographic parameters such as currents, stratification, oxygen concentration, and fluorescence, along with water-column backscattering from a multi-frequency echosounder. We revisit a series of relationships often considered in the literature but not always precisely evaluated. First, deep chlorophyll maximum (DCM) near-ubiquitous in stratified surfaces is classically associated with the thermocline/pycnocline, but precise evaluations are scarce and sometimes contradictory. Second, we examine the relationships between the epipelagic SSLs, the stratification and the DCM. Indeed, various authors have associated SSLs with these features [25, 31–35] however, relationships are generally lacking or contradictory. Finally, we consider the full set of environmental parameters to determine the potential primary factors driving the vertical distribution of sound scattering biota in relation to thermohaline area, season, and diel period.

## 2. Methods

### 2.1. Data collection and processing

Data were collected during the two multidisciplinary surveys Acoustic along the BRAzilian CoaSt (ABRACOS) performed off Northeast Brazil (Fig 1) aboard the French R/V Antea in September—October 2015 (austral spring; ABRACOS 1; [36]) and April—May 2017 (austral autumn; ABRACOS2; [37]). The survey track consisted of rectangular transects along the shelf and adjacent oceanic area and radial transects around oceanic islands and seamounts (Fig 1). Hydrological measurements were performed at 96 stations, including the 52 stations (22 in spring; 32 in autumn) in locations with bottom depths higher than 300 m used in this study (Fig 1).

Conductivity, temperature, depth, fluorescence, and dissolved oxygen hydrographic profiles were acquired using a CTD Seabird SBE911+ from the surface down to 1000 m or ten meters above the bottom. Conductivity, temperature, pressure, and dissolved oxygen accuracies are 3 mS/m, 0.001°C, 0.7 dbar, and 0.09 ml.l^–1^, respectively. To minimize the dynamic errors between the casts, conductivity and dissolved oxygen measurements from the water samples collected during the ascent of the rosette were used to optimize the adjustment coefficients of the sensors. This step consists in optimizing the adjustment coefficients, minimizing the differences, by an iterative process, between the chemical measurements and the probe measurements. The Wetlabs® ECO-FLNTU fluorometer, optical sensor, measures the fluorescence emission in a small volume of water, offering a relative measurement of chlorophyll-a. The chlorophyll-a measurement was performed at 0 to 50μg/l with a sensitivity of 0.025μg/l.

The zonal and meridional components of the currents were measured using an ’Ocean Surveyor’ ship-mounted acoustic Doppler current profiler (SADCP) operating continuously at 75 kHz with a depth range of 15–700 m. For more information on ADCP data processing see Dossa et al. (2021). To obtain mean current velocity profiles for each station, we calculated the average currents (zonal and meridional components and the resulting current) values over a circle of 0.02° around each CTD profile. The vertical current shear (*S*^2^) was calculated according to $S^{2}={\left(\partial_{z}u\right)}^{2}+{\left(\partial_{z}v\right)}^{2}$ Where *u* and *v* are the zonal and meridional components, respectively (Johnston and Rudnick, 2009). To determine the stability of the water column, we used the Brunt Väisälä frequency (N^2^, the buoyancy frequency squared), calculated according to $N^{2}=\frac{-g}{\sigma_{0}}\frac{\partial_{\sigma_{z}}}{\partial_{z}}$, where *g* is the acceleration of gravity and *σ*_0_ is a reference density at 10 m [38, 39].

Acoustic data were acquired using four Simrad EK60 scientific echosounders operating continuously during transects and hydrological stations and connected to split-beam transducers working at 38, 70 and 120 kHz an operational range from the surface to 700, 500 and250m, respectively (Table 1). However, we used data only down to 650, 450 and 200 m for frequencies of 38, 70 and 120, respectively, in order to exclude low signal-to-noise regions. Echosounder calibration was performed according to [40] using a tungsten carbide sphere. The astronomical diel periods (sunrise, day, sunset, and night) were defined from the time and geographical position. Day stations were considered to the extend from one hour after sunrise to one hour before sunset, while the night stations were from one hour after sunset to one hour before sunrise.

**Table 1:** Echosounder configuration sets.

Frequency (kHz)	38	70	120
**Transducer model (SIMRAD)**	ES38B	ES70-7C	ES120-7C
**Beam width (deg)**	6.9	7.0	7.0
**Transmit power (W)**	1000	750	200
**Pulse duration (μs)**	512	512	512
**Ping rate (s** ^**-1**^ **)**	1	1	1

Acoustic data processing, including automatic extraction of different types of noise, such as transient noise, attenuated signal, impulsive noise (Ryan et al., 2015), and background noise [41], was achieved using the Matecho software tool (version 20191213V6; [42]). The bottom-line correction and the removal of multiple echoes in the water column were performed manually. Near-surface noise was avoided by excluding the signal from the transducer to a fixed distance of 10 m. Acoustic data were expressed in volume backscattering coefficient (s_v_ in m^-1^, [43]) with a threshold of -100 dB. To get the same vertical resolution as the CTD, a high-resolution echo-integration on 1 ping per 1 m high cell was applied on s_v_. Volume backscattering strength (Sv in dB re 1 m^-1^, [43]) data were averaged over 100 pings to obtain the Mean Volume Backscattering Strength (MVBS, in dB re m^-1^). The 100 pings sections considered here are those acquired just after the end of each CTD cast to limit noise related to propeller during the CTD operation and to ensure working on genuine day and night data since most CTD were mostly performed during twilight periods.

**Fig 2:**
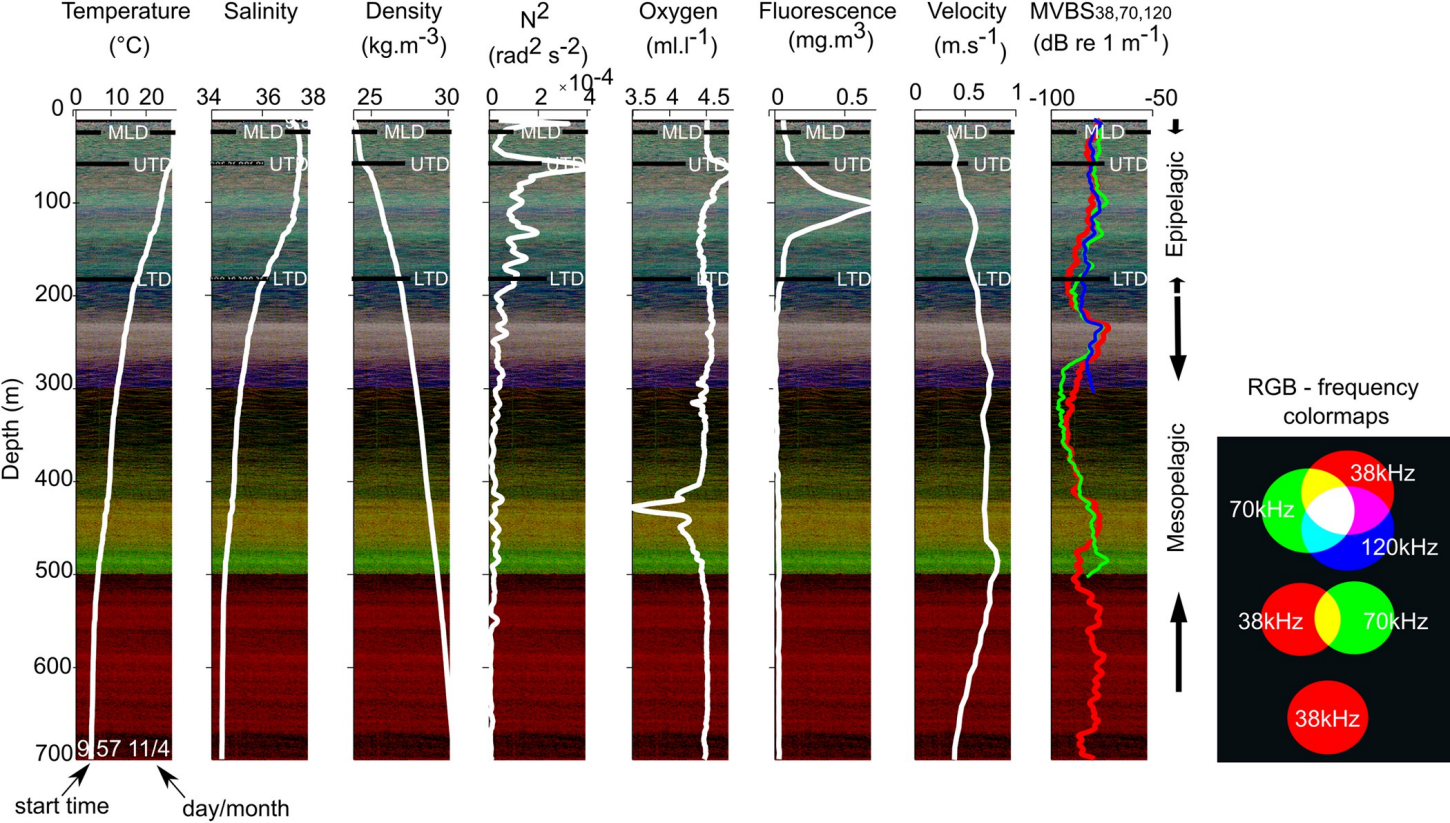
Example of RGB plot of 100 pings long echogram for station 06 of ABRAÇOS 2 (WBCS, day, see Fig 1C for the position) and corresponding vertical environmental profiles from CTD and ADCP data (solid white lines), as well as the MVBS at 38 (solid red line), 70 (solid green line) and 120 (solid blue line) kHz. MLD: mixed layer depth; UTD: upper thermocline depth; LTD: lower thermocline depth.

To visualize of the relative multifrequency acoustic information at each station, a Red Green Blue (RGB) composite image was generated and superimposed to the vertical profiles of the different physico-chemical parameters (Fig 2). To construct a RGB image, we rescaled the Sv from each frequency (38, 70, and 120 kHz) on a linear scale, which has 255 intervals from the minimal value (-100 dB) to the maximal one (-50 dB). This visualization technique allows highlighting the relative contribution of each frequency through the dominance of colour: red (38 kHz), green (70 kHz), blue (120 kHz). The colour thus indicates the dominant frequency, while the brightness approximates the average backscatter intensity of the three frequencies combined.

The biological samples, used as complementary data to support the discussions in this study, were collected at the same CTD stations during day and night (Fig 1). Zooplankton samples were collected using a Bongo net (60 cm of mouth diameter and mesh size of 300μm) obliquely to-wed from 200 m up to the surface. Mesopelagic fishes, crustaceans, and gelatinous organisms were collected using mesopelagic (body mesh 30 mm, cod-end mesh 4 mm) and micronekton (body mesh 40 mm, cod-end mesh 10 mm) nets from 10 to 1113m depth (here we work only up to 600 m) for about 30min at 2–3 knots. Targeted depth was defined for each trawl according to the presence of acoustic scattered layers or patches. Except the layers 200–300 at night, where no aggregation of organisms was observed through acoustics, all depth strata were sampled at least once. Tow duration was considered as the moment of the arrival of the net on the present depth to the lift-off time, recorded by means of a SCANMAR system. The net geometry was monitored using SCANMAR sensors providing headline height, depth, and distance of wings and doors. As the trawl did not have any opening or closing mechanism, the collection of specimens during the lowering or hoisting of the net was reduced as much as possible by decreasing ship velocity and increasing winch speed.

All collecting methods and specimen handling procedures were approved and carried out in accordance with relevant guidelines and regulations of the Brazilian Ministry of Environment (SISBIO; authorization number 47270–4 for ABRAÇOS 1 and 47270–5 for ABRAÇOS 2).

### 2.2. Data analysis

#### 2.2.1 Oceanscape

First, we describe the oceanscape, i.e., the vertical features of the environmental factors (currents, current shear, stratification, oxygen concentration and fluorescence) for each hydrodynamic system (WBCS and SECS) according to the season (spring and autumn). For that, we used a functional data analysis approach [44] to statistically test for seasonal differences in the vertical profiles of each environmental parameter in a given hydrodynamic system. This branch of statistics works on functions instead of discretized vectors to analyse the distribution and variability of data according to the physical dimension in which they are measured, the depth in our case. Discrete data profiles were transformed into a curve using a B-spline basis of degree 3 as smoothing [45]. B-splines are the most common system approach choices for non-periodic functional data. The number of basis functions (K) and the penalised parameter of the basis, which imply the degree of smoothing of the curve, was selected by the Generalised Cross-Validation (GCV) analysis performed using the R packages "fda", respecting the balance between smoothing and the desired scale (1 m) of variability [46]. Therefore, before performing GCV, we defined a range of potential K by performing a smoothing test for each variable individually according to different K. All environmental factors showed good smoothing within the range of 41–42 basis (K). At the same time, the acoustic profiles fitted best with 44 basis. The higher the number of K-functions, the more complexity is preserved. Once this was done, we used the functional analysis of variance (fANOVA), which uses all the information of each mean functional curve to test for differences based on the shape and spatial (along with depth) variability of the curves [27, 44, 47]. The significance testing value (p-value) considered in this study was 0.05.

Vertically, we divided the acoustic data into two layers: (i) the epipelagic layer, defined from 20 to 200 m; and (ii) the mesopelagic layer, from 200 to 650 m. This choice was made considering the thermohaline structure in the region with the most thermohaline gradients restricted to the first 200 m [27] and the scrutinising of the most recurrent SSLs in RGB echograms (Fig 2). We applied a fANOVA approach to compare the shape of the acoustic profiles at each frequency in each hydrodynamic system and layer according to the season and the diel cycle.

#### 2.2.2. Cross-correlations

To study the potential relationships between the fluorescence profiles and the stratification and between the acoustic profiles and each environmental factor (stratification, fluorescence, dissolved oxygen and currents) along with the depth, we used the cross-correlations function (CCF, [48]). The CCF helps identify vertical shifts (*h* in metres) of the *x* (environmental factor) variable that might be valuable predictors of *y* (MVBS in dB re 1 m^−1^). In R, the CCF is defined as the set of sample correlations between *x_d+h_* and *y_d_* for h = 0, ±1, ±2, ±3, and so on. For instance, a negative value for *h* correlates the *x* variable at a depth before *d* and the *y* variable at depth *d*. The cross-correlation analyses were done for the epipelagic and mesopelagic layers, separately.

**Fig 3:**
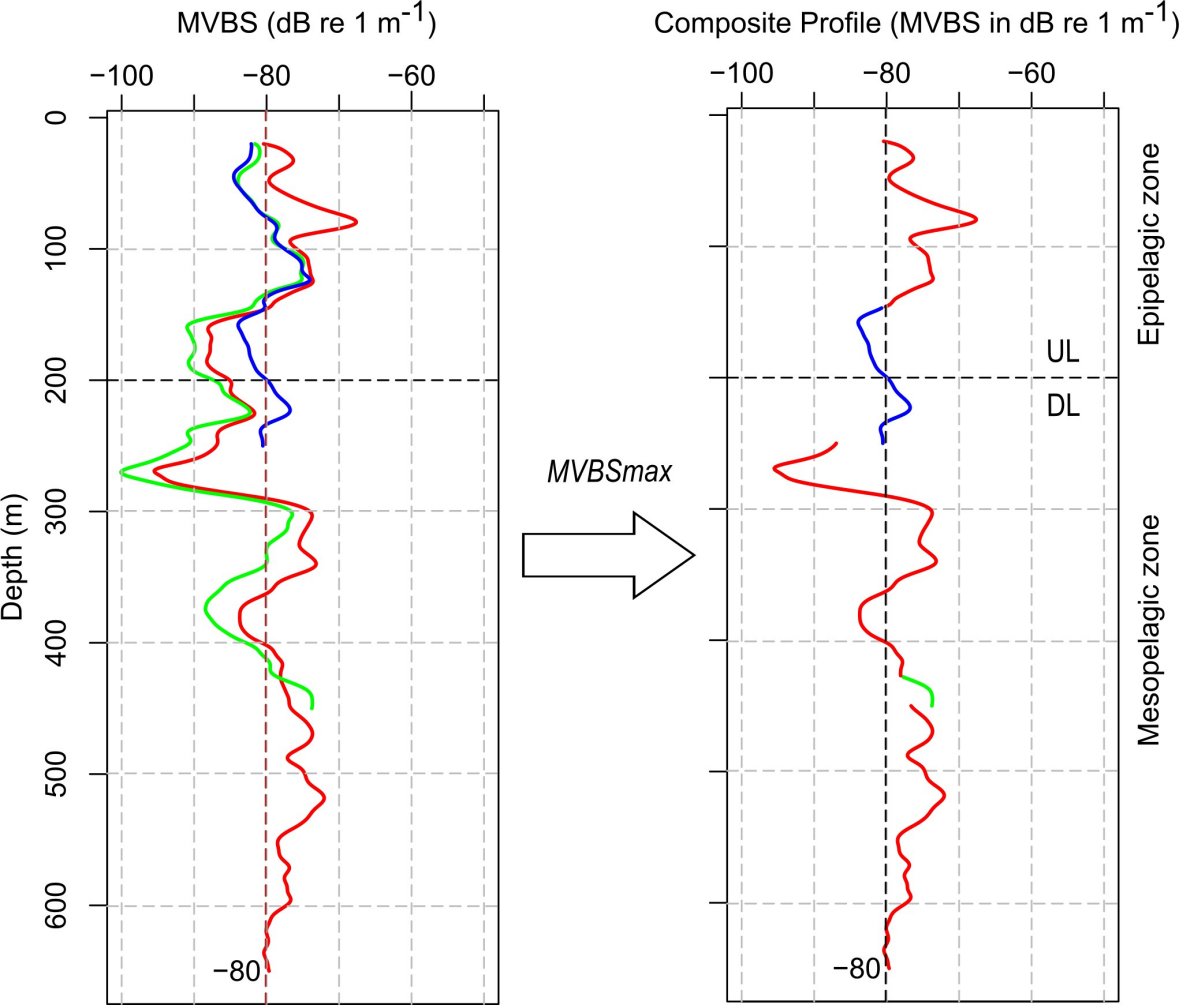
Example of three MVBS acoustic profiles at 38, 70, and 120 kHz and resulting composite profile.

In addition, considering the different factors we explore (hydrodynamic system, layer, season, and diel period), addressing each frequency separately led to too many tests increasing the risk of stochastic results. Although we performed the correlations for each frequency separately, we focused on the maximum acoustic response between these three frequencies at each depth in order to synthetize the information from the three frequencies. For that purpose, we constructed a composite profile (MVBS, in dB re m^-1^) that was derived from the three frequency profiles (38, 70, and 120 kHz), in which for each depth, we used the highest value across the three frequencies analysed (Fig 3).

Finally, for a comprehensive description of the structure of the acoustic profiles, we defined the SSLs as the depth range where the composite profiles exceeded the overall median value of the acoustic backscatter in each vertical zone (epipelagic or mesopelagic), in a given hydrodynamic system (WBCS or SECS), season (spring or autumn) and diel period (day or night). See Table 2 for more details.

**Table 2:** Median composite profile values (in dB re m^-1^) adopted as the threshold to define the SSLs in each season and zone (EP–epipelagic; MP–mesopelagic). These thresholds correspond to the averages of the values of the stations sampled according to each condition (season, diel period, and system).

		Western boundary current system—WBCS		South equatorial current system—SECS	
** **		**Day**	**Night**	**Day**	**Night**
**Spring 2015**	**EP**	-82	-77	-81	-78
	**MP**	-87	-88	-82	-82
**Autumn 2017**	**EP**	-82	-76	-80	-79
	**MP**	-86	-86	-83	-83

## 3. Results

### 3.1. Oceanscape

**Fig 4:**
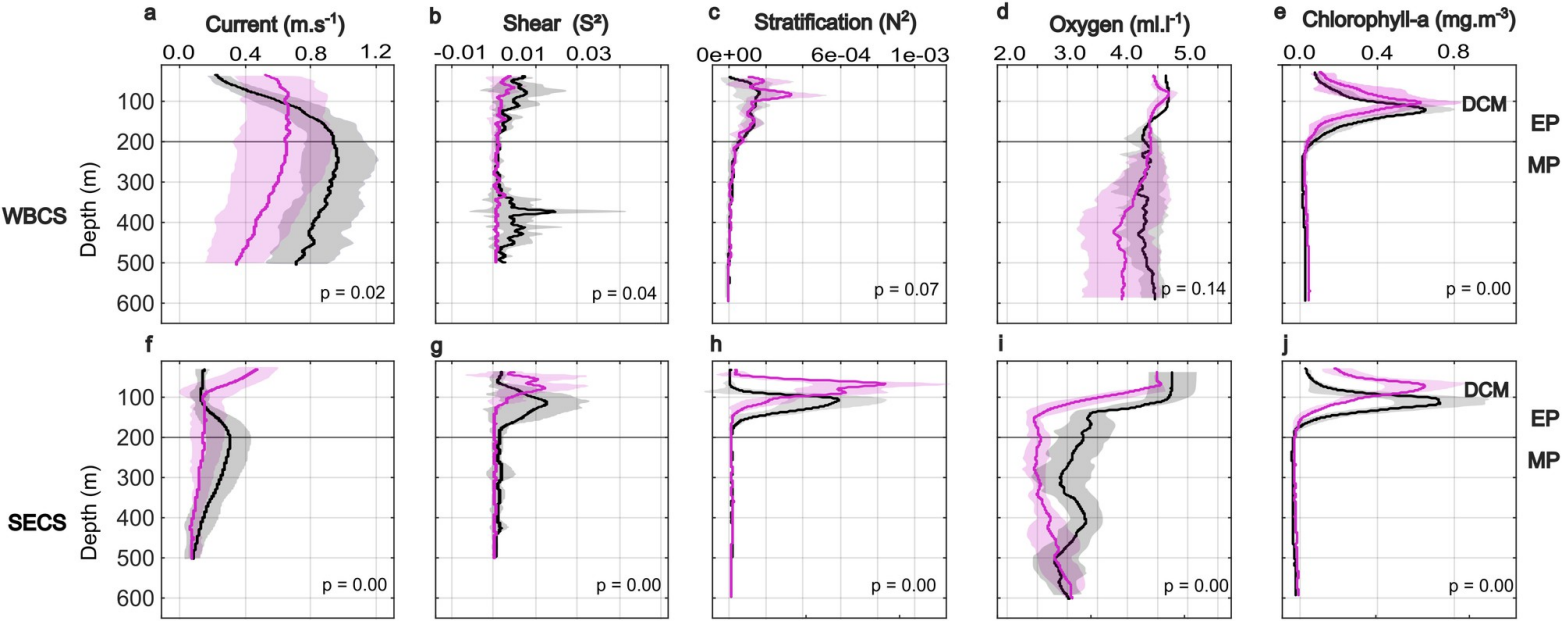
Mean profiles of the current velocity (a, f), shear (b, g), stratification (c, h), dissolved oxygen (d, i), and chlorophyll-*a* with an indication of the deep chlorophyll maximum–DCM (e, j) in spring of 2015 (black) and autumn of 2017 (magenta) in the western boundary current system (WBCS, a-e) and the south equatorial current system (SECS, f-j). The range of ADCP data (current and shear) was limited to 500 m deep. Shaded areas show the standard deviation of the profiles. The epipelagic (EP; above 200 m) and mesopelagic (MP; below 200 m) zones are represented.

In the Western Boundary Current System (WBCS), 3 out of 5 parameters presented significant seasonal differences (Fig 4). The undercurrent, NBUC, was significantly more intense in spring than in autumn, particularly between 140–320 m (Fig 4A). Current shear (S^2^, Fig 4B) was also considerably more intense in spring, especially in the range 40–140 m (thermocline/pycnocline) and 320–450 m (no shearing observed in autumn). Even if the overall difference was not significant (p = 0.07), the stratification (N^2^, Fig 4C) was lower in spring than in autumn. The entire water column in the WBCS region was well oxygenated whatever the season (mean values between 3.8 and 5 ml.l^-1^), with, however, a layer with moderate deoxygenation (~3.2 ml.l^-1^) at 300–500 m in autumn (Fig 4D). Finally, the deep chlorophyll maximum (DCM, Fig 4E) was shallower (~90 vs 100 m) and slightly stronger in spring than in autumn.

In the South Equatorial Current System (SECS, see Fig 1), which encompasses the oceanic islands and seamounts, all parameters varied significantly according to the season (Fig 4). Westward surface current (cSEC, down to 100 m) was stronger in autumn, while the eastward subsurface current (SEUC) was much more intense in spring (Fig 4F). Current shear (S^2^, Fig 4G) between the surface and subsurface opposing currents was stronger and deeper in spring (~90–120 m) than in autumn (~40–60 m). The thermocline/pycnocline (maximum stratification (N^2^) layer, Fig 4H) was also deeper (~90 vs 50 m) in spring than in autumn. Unlike the WBCS, the SECS presented a less oxygenated layer below the thermocline/pycnocline with lower values in autumn (2.4 ml.l^-1^) than in spring (2.8 ml.l^-1^) (Fig 4I). Finally, the DCM (Fig 4J) was deeper (~100 vs 50 m) and stronger in spring than in autumn.

### 3.2. Epipelagic layer

**Fig 5:**
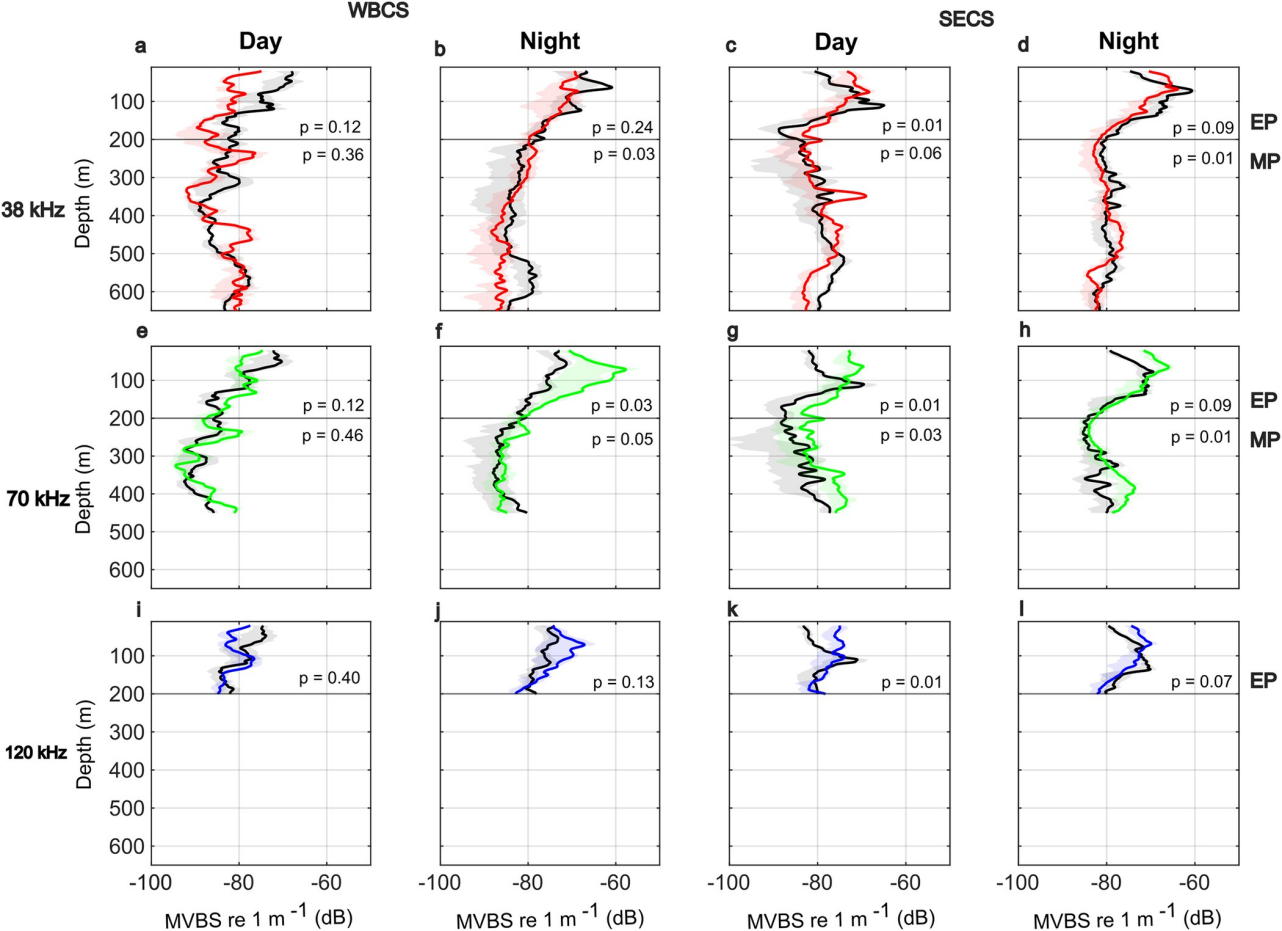
Functional ANOVA between the mean acoustic profiles (MVBS dB re m^-1^, continuous line) with the shaded areas indicating an interquartile range (25, 75 percentiles) by frequency (38, 70 and 120 kHz), diel period (day; night), hydrodynamical system (WBCS; SECS), layer (EP–epipelagic; MP–mesopelagic) and season: Spring 2015 (black lines) and autumn 2017 (coloured lines).

In the epipelagic layer, SSLs were omnipresent throughout the SWTA, varying in intensity (proxy of biomass) and vertically, according to the diel period, season (spring and autumn), and the hydrodynamic system (WBCS and SECS). The MVBS was stronger at night, whatever the system, season and frequency (Fig 5; S1 Fig). In the WBCS, the MVBS decreased with depth whatever the diel period, season and frequency except at 70 and 120 kHz at night in autumn, when it presented a maximum at ~70 m. In the SECS, the pattern was different since the MVBS always peaked in the sub-surface (at ~70–120 m) except at 70 and 120 kHz during the day in autumn, when it decreased with depth (Fig 5).

**Fig 6:**
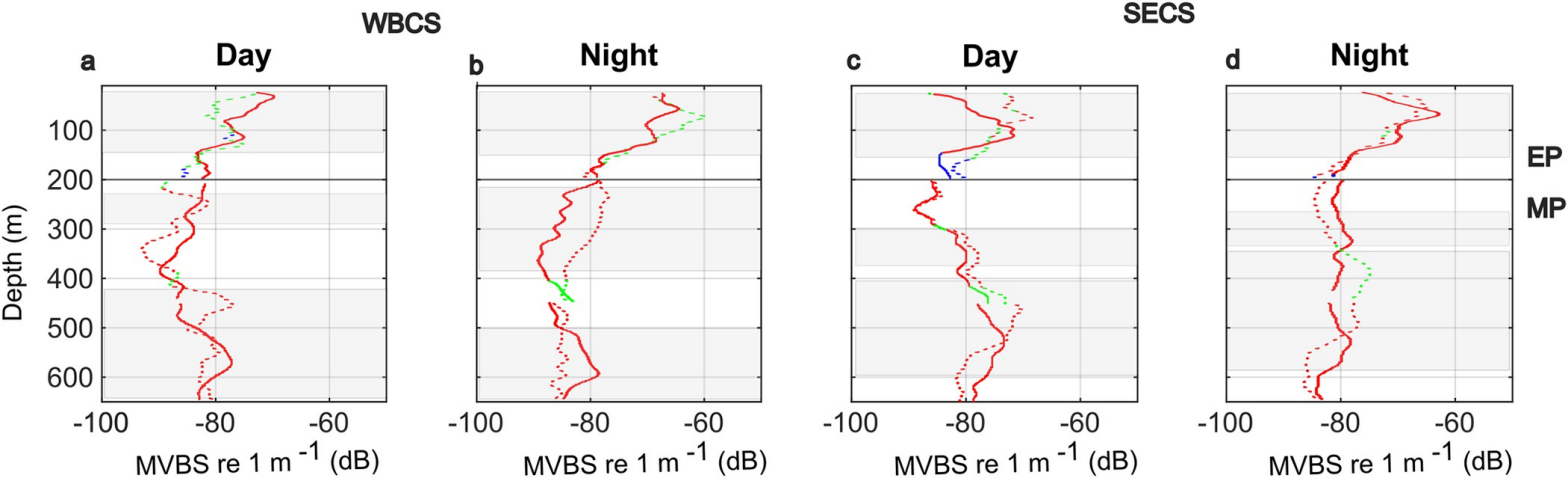
Mean Composite profile (MVBS, dB re m^-1^) acoustic profiles in spring 2015 (continuous lines) and autumn 2017 (dashed lines) for each diel period (day; night) and hydrodynamical system (WBSC; SECS). The respective profiles are composed of the frequency (38 kHz in red, 70 kHz in green, and 120 kHz in blue), providing the highest backscatter at each depth. The compositions are divided between the epipelagic layer (EP; above 200 m) and the mesopelagic layer (MP), where only 38 and 70 kHz are available. Main SSLs are highlighted in light grey.

The profiles of maximum acoustic response (composite profile) allow better observation of how the dominant backscatter varies according to the depth (Fig 6). In spring, 38 kHz was the dominant frequency in the epipelagic layer, whatever the diel period and system. The only exception was in the SECS during the day, with the MVBS120 dominating below 150 m deep. In autumn, the feature was different, with an overall dominance of MVBS70 in the WBCS at both diel periods. In the SECS, MVBS38 dominated at night, while during the day we observed an alternation between MVBS38 and MVBS70 in the first 160 m, then the dominance of MVBS120.

### 3.3. Cross-correlations in the epipelagic layer

Here we describe the results of the cross-correlations between composite profile and environmental factors. For cross-correlations with individual frequencies for both epipelagic and mesopelagic zones see S1 Table.

**Fig 7:**
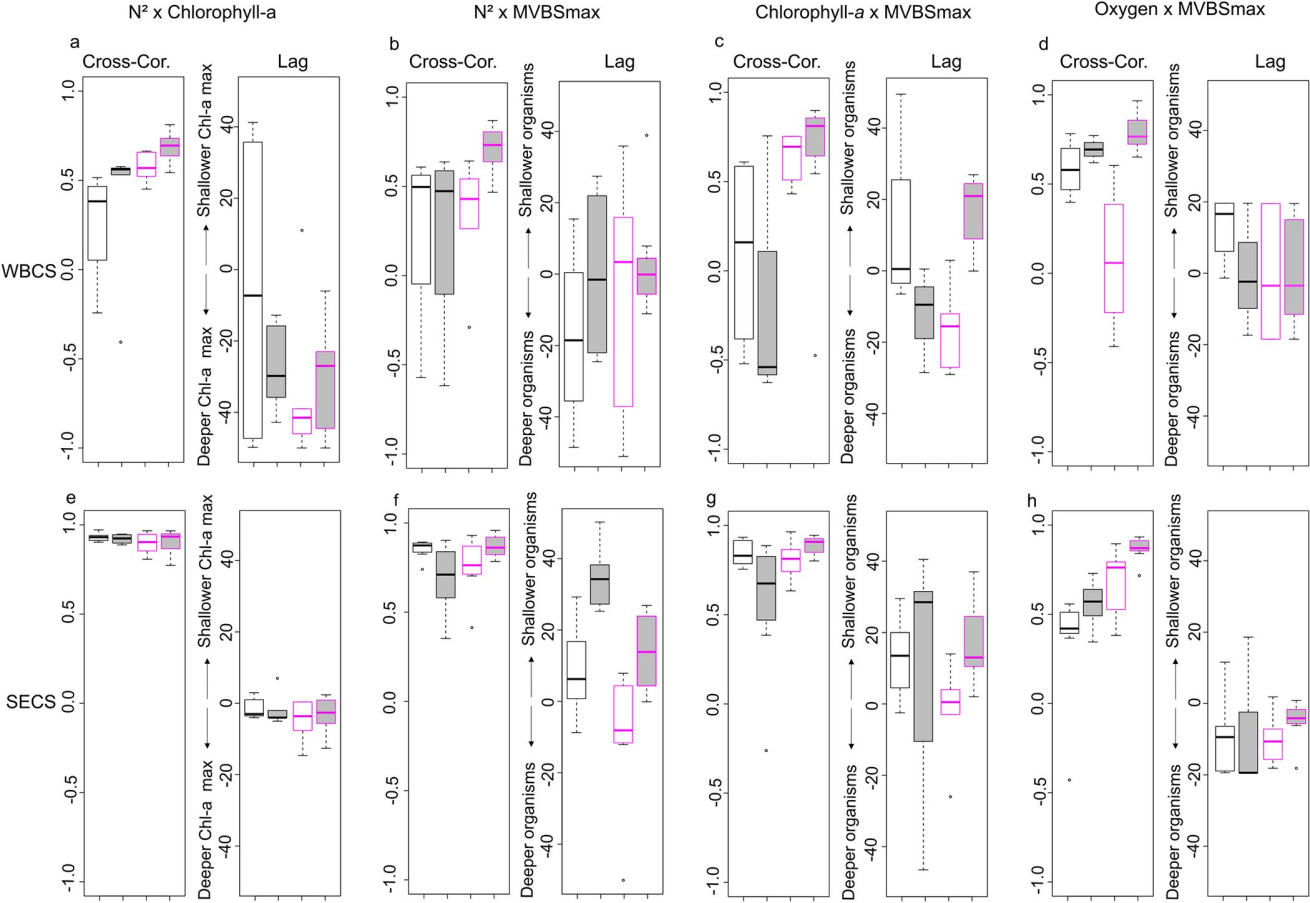
Boxplots of the epipelagic cross-correlations (Cross-Cor.), the vertical shift of maximum correlation, between the (a, e) stratification (N^2^) and chlorophyll-*a* (mg. m^-^^3^), and also between the composite profile profiles and the (b, f) stratification, (c, g) chlorophyll-*a* (mg. m^3^) and (d, h) oxygen (ml.l^-1^). All analyses are presented separately for each hydrodynamic system (WBCS; SECS) in relation to seasons (spring: black; autumn: pink) and diel period (day: white; night: grey). The correlations by individual frequency are shown in S1 Table.

In all cases, chlorophyll-*a* was positively correlated with stratification. The strength of the correlation was higher in the SECS (above 0.9) than in the WBCS (~0.4 to 0.7). In the SECS, the DCM matched (vertical shift ~0) the maximum stratification (Fig 7D). However, in the WBCS, the DCM was ~30 to 40 m below the maximum stratification (S2 Fig), except in spring at day when the relationship was weaker (Fig 7A).

As for the DCM, the composite profile was positively correlated to the maximum stratification but to a greater extent in the SECS (Fig 7B and 7F). In the WBCS (Fig 7B), the peaks of composite profile matched the depth of maximum stratification except in spring at the day when it was ~20 m below. In the SECS (S3 Fig) in autumn, both peaks matched at day, but the peak of composite profile was above the maximum stratification at night.

Backscattering levels of epipelagic SSLs were generally positively correlated with high oxygen concentrations (Fig 7D and 7H), except in autumn 2017 at day, when the correlation was close to null. In the WBCS (Fig 7D), no vertical shift was observed between the SSLs and the maximum oxygen concentration, but in spring during the day when SSLs were shallower (~18 m) than maximum oxygen concentrations. In the SECS (Fig 7H), on the other hand, SSLs were deeper than oxygen maxima, with mean vertical shifts ranging between 5 and 20 m.

### 3.4.Mesopelagic layer

At the mesopelagic zone, a SSL was observed in most of the stations (45 out of 54) (Fig 5). SSLs number, thickness, vertical position, and intensity varied according to the hydrodynamic system, the season and the diel period. In the WBCS, at day, MVBS profiles at 38 and 70 kHz did not vary significantly according to the season (Fig 5A and 5E). The main feature was the presence of SSL at ~250 m and below 450 m. At night (Fig 5B and 5F), the seasonal difference was significant with higher intensity and more preeminent SSL in the range 200–350 m in autumn. In spring, however, a conspicuous SSL was present in the range 500–650 m depth during the day. In the SECS (Fig 5C, 5D, 5G and 5H), the vertical MVBS at 38 and 70 kHz varied significantly according to the season except at 38 kHz at day (p = 0.06). During the day, the main pattern was an SSL in the range of 400–500 m. At night, no strong patterns were observed at 38 kHz, while a pronounced SSL was observed at 70 kHz in autumn at 350–450 m.

### 3.5. Cross-correlation in the mesopelagic layer

**Fig 8:**
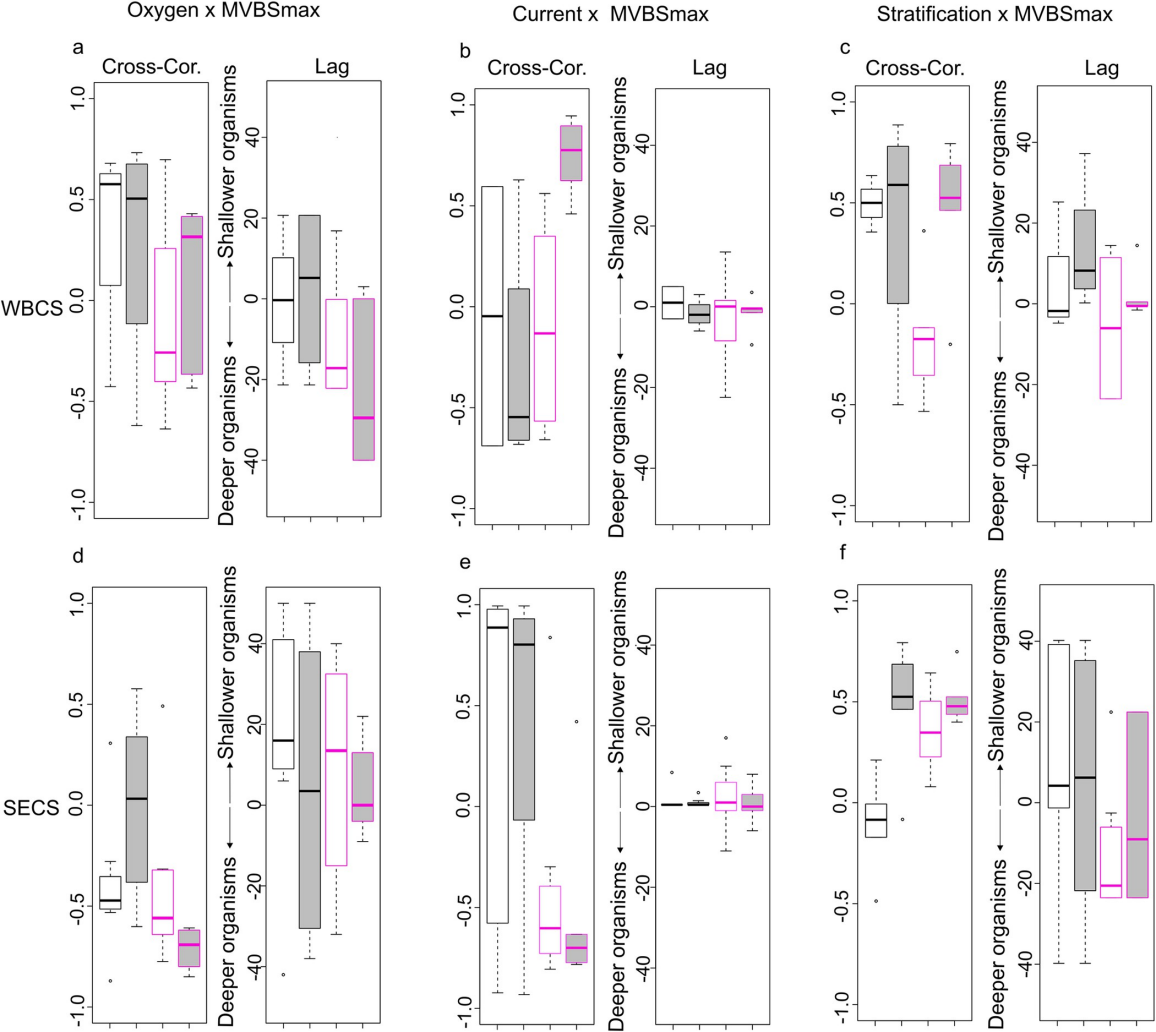
Boxplots of the mesopelagic cross-correlations (Cross-Cor.) and the vertical shift of maximum correlation between the Composite profile profiles and the (a, d) oxygen (ml.l-1), (b, e) current (m.s^-1^), and (c, f) stratification (N^2^). All analyses are presented separately for each hydrodynamical system (WBCS; SECS) in relation to seasons (spring—black; autumn—pink) and diel period (day—white; night—grey). The correlations by individual frequency are shown in S1 Table.

In the mesopelagic layer, the cross-correlation between composite profile and oxygen concentration varied according to the season, the system and the diel period (Fig 8A and 8D). In the WBCS, where less impact of oxygen concentration is expected, because the whole water column along the WBCS system is well oxygenated ([OD]>3ml.l^-1^), a weak positive correlation with no vertical shift was observed in spring. In the autumn, the patterns were less clear, with a lower correlation associated with high variability. In the SECS, a clear negative correlation was observed (mean upward vertical shift ranging from ~0 to 20 m), except in spring at night.

The cross-correlation between composite profile and resulting currents varied according to the season, the system and the diel period (Fig 8B and 8E). In the WBCS, no correlation was observed except at night in autumn, when a strong positive correlation with no vertical shift was observed. In the SECS, positive and negative correlations with no vertical shift were observed in spring and autumn, respectively.

Finally, the cross-correlation between Composite profile and the stratification also varied according to the season, the system and the diel period (Fig 8C and 8F). In the WBCS, a positive correlation with vertical shift close to 0 was observed in all cases except in autumn at the day when negative. In the SECS, a positive correlation was observed in all cases except in spring 2015 at day when no correlation was observed. The vertical shift was close to 0 except in autumn at day when it was negative (~20 m).

### 3.6. Biological catches composition

The vertical distributions of organisms by major groups, were observed considering depth intervals from the surface to 200 m (epipelagic zone) and every 100 m to 600 m (Tables 3 and 4). The different dial periods (day and night), the sampling stations, the respective hydrodynamic systems, and the seasons (spring and) were considered separately.

**Table 3:** Species collected from daytime stations, survey (S: spring—ABRACOS 1; F: autumn—ABRACOS 2), number of specimens (N°), site (Western Boundary Current System–WBCS; South Equatorial Current System—SECS), depth range. This catalogue has been constructed, in summary, based on the ABRACOS project database. The expressions in bold indicate the locations or seasons with the greatest abundance. Please, for further information, see the references mentioned.

REFERENCES	FILO	CLASS	ORDER	FAMILIES	NUMBER	AREA	SEASON
**< 200 m**							
	**Mollusca**	Cephalopoda			36	WBCS—**SECS**	S—**F**
10.1016/j.jmarsys.2020.103449	**Arthropoda (Crustacea)**	Malacostraca	Euphausiacea		192	WBCS—**SECS**	S
			Decapoda		22	**WBCS**—SECS	S
			Amphipoda		5	WBCS	S
10.1093/plankt/fbaa066	**Cnidaria (BV)**				255.5	WBCS—**SECS**	S—**F**
	**Chordata (Tunicata)**	Thaliacea (bv)	Salpida	Salpidae	17	WBCS—**SECS**	S
			Pyrosomida	Pyrosomatidae	279	WBCS	F
			Doliolida		26	SECS	F
	**Chordata**	Actinopterygii	Pleuronectiformes	Bothidae	180	SECS	S
10.1016/j.pocean.2021.102695			Myctophiformes	Myctophidae	548	WBCS—**SECS**	S—**F**
10.1016/j.pocean.2020.102389			Stomiiformes	Sternoptychidae (Hatchetfishes)		**WBCS**—SECS	**S**—F
				Gonostomatidae	36	SECS	S—**F**
10.1038/s41598-020-77222-8				Stomiidae			
10.15560/15.6.965 and 10.1080/17451000.2019.1636281			Perciformes		37	SECS	S—**F**
10.1080/17451000.2021.1891806			Teleostei		24	SECS	F
**200–300 m**							
10.1016/j.pocean.2021.102695	**Chordata**	Actinopterygii	Myctophiformes	Myctophidae	54	WBCS	F
10.1016/j.pocean.2020.102389			Stomiiformes	Sternoptychidae (Hatchetfishes)	14	WBCS	F
10.1080/17451000.2021.1891806			Teleostei		27	WBCS	F
	**Ctenophora (BV)**				60	WBCS	F
	**Jellyfish (BV)**				143	WBCS	F
**300–400 m**							
10.1016/j.pocean.2021.102695	**Chordata**	Actinopterygii	Myctophiformes	Myctophidae	56	SECS	F
10.1016/j.pocean.2020.102389			Stomiiformes	Sternoptychidae (Hatchetfishes)	101	SECS	F
				Gonostomatidae	67	SECS	F
	**Cndaria (colonies)**			Siphonophera	5	SECS	F
**400–500 m**							
	**Cndaria (BV)**				404	WBCS	F
	**Ctenophora (BV)**				23	WBCS	F
	**Jellyfish (BV)**				20	WBCS	F
	**Chordata (Tunicata)**	Thaliacea	Pyrosomida (BV)	Pyrossomidae	217	WBCS	F
	**Arthropoda (Crustacea)**	Malacostraca	Euphausiacea		7	WBCS	F
			Decapoda		32	WBCS	F
	**Chordata**	Actinopterygii	Stomiiformes	Gonostomatidae	39	WBCS	F
10.1016/j.pocean.2020.102389				Sternoptychidae (Hatchetfishes)	5	WBCS	F
**500–600 m**							
	**Arthropoda (Crustacea)**	Malacostraca	Euphausiacea		515	SECS	S
			Decapoda		21	WBCS	S
			Amphipoda		89	SECS	S
			Stomatopoda (Latreille, 1817)		54	SECS	S
	**Cnidaria (BV)**				85.5	WBCS—**SECS**	S
	**Chordata (Tunicata)**	Thaliacea	Pyrosomida	Pyrossomidae	5	WBCS	S
			Doliolida		10	SECS	S
			Salpida	Salpidae	50	SECS	S
10.1016/j.pocean.2021.102695	**Chordata**	Actinopterygii	Myctophiformes	Myctophidae	49	WBCS—**SECS**	S
10.1016/j.pocean.2020.102389			Stomiiformes	Sternoptychidae (Hatchetfishes)	24	**WBCS**—SECS	S
				Gonostomatidae	54	**WBCS**	S
10.15560/15.6.965 and 10.1080/17451000.2019.1636281			Perciformes	Decapterus (Carangidae)	22	WBCS	S

**Table 4:** Species collected from night stations, survey (S: spring—ABRACOS 1; F: autumn—ABRACOS 2), number of specimens (N°), site (Western Boundary Current System–WBCS; South Equatorial Current System—SECS), depth range. This catalogue has been constructed, in summary, based on the ABRACOS project database. The expressions in bold indicate the locations or seasons with the greatest abundance. Please, for further information, see the references mentioned.

REFERENCES	FILO	CLASS	ORDER	FAMILIES	NUMBER	AREA	SEASON
**< 200 m**							
	**Mollusca**	Cephalopoda	Oegopsida		113	**WBCS**—SECS	S—**F**
			Octopoda				
10.1016/j.jmarsys.2020.103449	**Arthropoda (Crustacea)**	Malacostraca	Euphausiacea		1004	**WBCS**—SECS	S
			Decapoda		83	SECS	S
			Stomatopoda		354	**WBCS**—SECS	S
10.1093/plankt/fbaa066	**Cnidaria (BV)**				1333	WBCS	S-**F**
	**Chordata (Tunicata)**	Thaliacea (bv)	Salpida	Salpidae	287	WBCS—**SECS**	S-**F**
			Pyrosomida	Pyrosomatidae	1469	WBCS	S-**F**
			Doliolida		25		F
	**Chordata**	Actinopterygii	Pleuronectiformes	Bothidae	169	WBCS—**SECS**	S
10.1016/j.pocean.2021.102695			Myctophiformes	Myctophidae	82	WBCS—**SECS**	S
10.1016/j.pocean.2020.102389			Stomiiformes	Sternoptychidae (Hatchetfishes)	43	WBCS—**SECS**	F
				Gonostomatidae			
10.15560/15.6.965 and 10.1080/17451000.2019.1636281			Perciformes		89	**WBCS**—SECS	S—**F**
			Teleostei		82	WBCS	S—**F**
			Aulopiformes		358	WBCS—SECS	S-F
			Tetraodontiformes	Molidae	20	WBCS	S
	**Mollusca**	Cephalopoda	Oegopsida		14	SECS	F
	**Chordata**	Actinopterygii	Stomiiformes	Gonostomatidae	30	SECS	F
10.1016/j.pocean.2020.102389				Sternoptychidae (Hatchetfishes)	55	SECS	F
			Beryciformes		15	SECS	F
**500–600 m**							
	**Arthropoda (Crustacea)**	Malacostraca	Euphausiacea		337	SECS	S
			Decapoda		120	SECS	S
			Stomatopoda (Latreille, 1817)		6	SECS	S
10.1016/j.pocean.2021.102695	**Chordata**	Actinopterygii	Myctophiformes	Myctophidae	220	SECS	**S**—F
10.1016/j.pocean.2020.102389			Stomiiformes	Sternoptychidae (Hatchetfishes)	65	SECS	S—**F**
				Gonostomatidae	149	**WBCS**—SECS	**S—**F
			Pleuronectiformes	Bothidae	211	SECS	S
			Labriformes		37	SECS	S
			Stephanoberyciformes	Melamphaidae	31	SECS	S

## 4. Discussion

**Fig 9:**
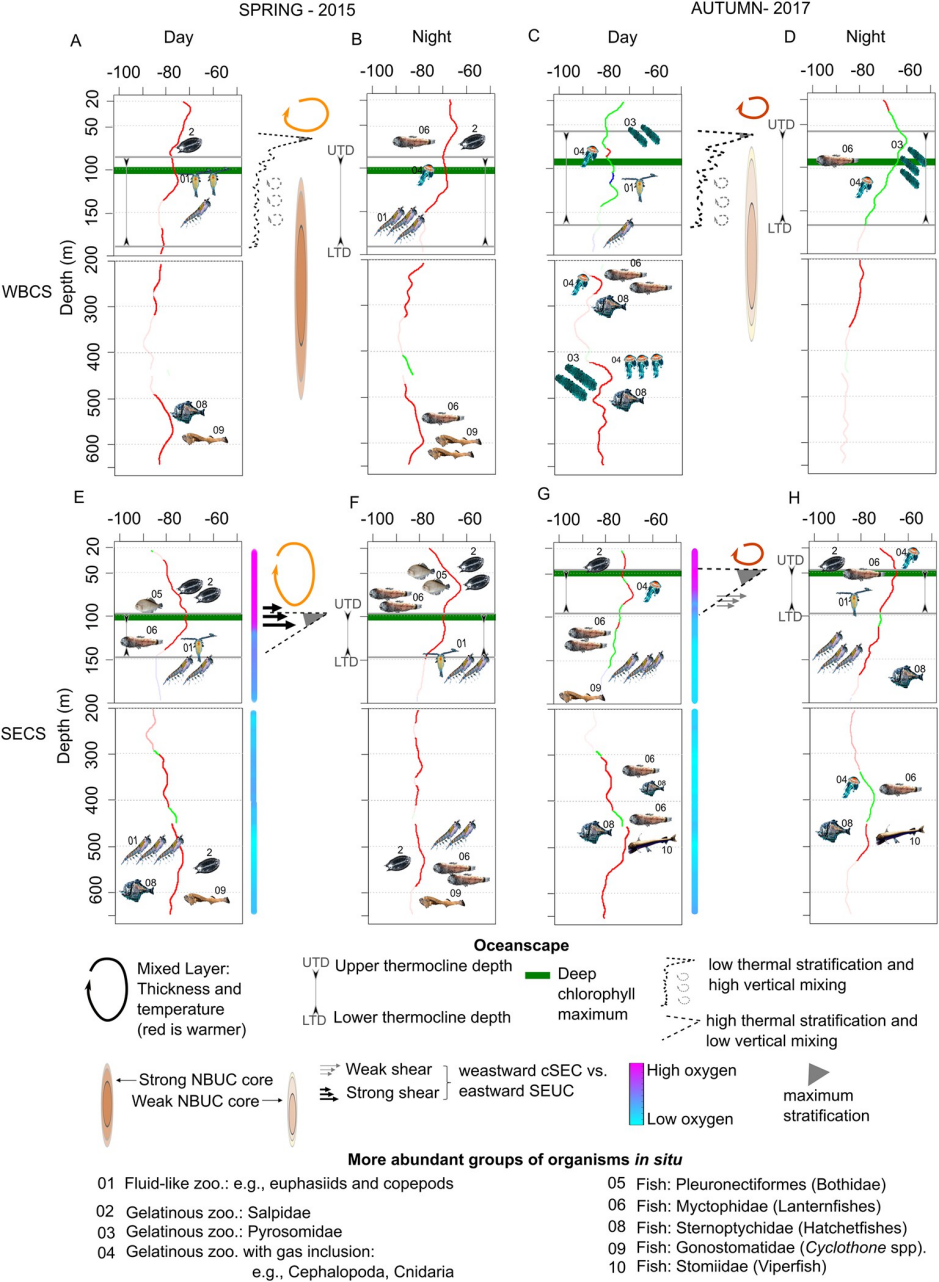
Schematic representation of the main environmental factors impacting the vertical distribution of the Sound Scattering Layers (SSLs) in spring 2015 and autumn 2017 in the western boundary current system (WBCS) and the south equatorial current system (SECS) according to the diel periods. The mean composite profile acoustic profiles (in dB re m^-1^) composed by the frequency (38 kHz in red, 70 kHz in green, and 120 kHz in blue) presenting the highest backscatter at each depth are shown in each panel. In these profiles, the depth sections with thick lines correspond to identified SSLs. The epipelagic zone (above 200 m) and the mesopelagic zone where only 38 and 70 kHz are available are considered separately.

Here we used multi-frequency acoustic, CTD, and ADCP data to describe the fine-scale vertical relationships between environmental factors and the vertical distribution of the backscattering acoustic in two seasons and two hydrodynamic systems of the SWTA according to the diel cycle. We build conceptual models relating the more robust relationships between the characteristics of SSLs and the environmental variables (Fig 9). We asked the environmental variables that showed these more robust correlations "potential environmental drivers" of bottom-up structuring. We propose four scenarios for both the WBCS (Fig 9A–9D) and the SECS (Fig 9E–9H), referring to seasons (spring and autumn) and diel periods (day and night). These models are based on the results obtained from this work and complemented with information from species distribution databases of the ABRACOS surveys (Tables 3 and 4).

Epi- and mesopelagic SSLs were omnipresent at both diel periods and seasons. Besides the expected increase in epipelagic acoustic backscatter at night due to the DVM, the patterns of SSLs vertical distribution varied according to each oceanscape (Fig 9). Here, even if the upper and mesopelagic layers are connected through the DVM (e.g., [22, 49, 50]), we discuss the main results considering the epipelagic and mesopelagic layers separately.

### 4.1. Epipelagic layer

According to the bottom-up structuring framework, physics drives the primary productivity, which in turn causes the distribution of organisms both horizontally and vertically (e.g., [2, 51]). First, we evaluate the relationships between the thermohaline stratification (N^2^) and the primary productivity chlorophyll-*a*. Although DCM was positively correlated with stratification (Fig 7), the form of the relationships was modulated by the thermohaline structure of the hydrodynamic system. In the SECS where strong, thin, and stable thermocline occurs, the correlation was remarkably high, and the DCM usually matched (no vertical shift) the peak of maximum stratification. Interestingly, this relationship was not affected by the strong seasonal modulation of the thermohaline structure with a thermocline ~40 m deeper in spring than in autumn (Figs 4 and 9E–9H). On the contrary, in the WBCS where the stratification is feebler and associated with a less pronounced and thicker thermocline, the correlation between the DCM and the peak of maximum stratification was weak (Figs 7B, 9A and 9B). In addition, in this case, the DCM was ~10 to 40 m deeper than the layer of maximum stratification. The maximum stratification was generally not associated with the thermocline (S2 Fig). Instead, it was related to the base of the mixed layer where a barrier layer occurs [27]. However, instead of the maximum stratification, the DCM was more associated with the thermocline (Fig 9A and 9D), which lies deeper than the pycnocline, in coherence with the nutricline [52]. Thus, whatever the season or the diel period, the vertical shift between the depth of maximum stratification and the DCM was more related to the spatial modulations of the barrier layer thickness (see [27]) than to other parameters.

In a second step, we examine the relationships between the epipelagic SSLs (composite profile) the stratification and the DCM. In the SECS, where the correlation between the peak of stratification and the DCM was extremely high and almost not vertically lagged, it was not possible to fully discriminate the impact between these two parameters (Fig 7F and 7G). We observed a strong correlation with low seasonal difference in both cases but an apparent diel effect in the vertical shift (Figs 7F, 7G and 9E–9H). During the day, the vertical shift between stratification and maximum backscatter was ~0, while at night, the peak of acoustic density was ~10 to 40 m above the one of stratification. According to [31], DCM was likely to be found above the zooplankton layer as below, indicating that the factors driving those relationships likely vary amongst systems and species. However, [17] observed that fine acoustic layers of zooplankton were always ~1 m deeper than the DCM. They attributed this negative offset to specific physical forcing or a foraging tactic with predators attacking beneath. At both diel periods and seasons, the composite profile was dominated by the 38 kHz (Fig 9E–9H), likely hiding organisms with “fluid-like” acoustic properties, which usually show an increase in backscatter with an increase in frequency [9, 53, 54]. The 120 kHz peaks (Fig 4), is probably more representative of crustacean zooplankton, occurred below the thermoclines (Fig 8E–8H).

In spring (Fig 9E and 9F), the trawl data indicate a conspicuous layer of salps dominated the mixed layer (Tables 3 and 4), these gelatinous organisms usually can be detected at frequencies of 120 and 38 kHz [55]. Salps have higher per-individual filtration rates of all the marine zooplankton filter feeders, being able to feed on pico- and nanophytoplankton [56, 57] that are less dependent on nutrients and can flourish above the DCM. In addition, many gelatinous are susceptible to degradation of colonies (mainly the small colonies) when exposed to regions of high vertical shear stress, as observed in the SECS spring 2015, tending to avoid areas like these, which may further explain their position within the mixed layer [58]. In the SECS, the reduction of the acoustic energy with depth also matches the profiles of dissolved oxygen (Figs 4, 8E–8H), organisms may indeed avoid the subsurface layer where dissolved oxygen can be close to 2 ml.l^-1^, a value that is known to restrict the distribution of many organisms (e.g., [59]). Interestingly the positive correlation with dissolved oxygen was higher at night than at day. As discussed later, this could be related to the ’oxygen debt’ some migrant organisms need to pay (see [60]).

In the WBCS, epipelagic SSLs were below the peak of stratification during the day and at a similar depth at night (Fig 8A and 8B). On the contrary, in the autumn, the correlation was stronger with the DCM with a negative vertical shift during the day and a positive vertical shift at night (Fig 9C and 9D). In both cases, the trend in vertical shift matched the diel vertical migration of organisms. In autumn 2017 (Fig 9C and 9D), pyrosomes (*Pyrosoma atlanticum*; Tables 3 and 4) were highly abundant in the WBCS [61]. Their frequency response was maximum at 70 kHz in the surface layer in autumn (Figs 5 and 6). Pyrosomes diel migrate and primarily forage on phytoplankton [62, 63], which may explain the stronger association with chlorophyll-*a* than stratification in autumn. Another factor in this region is the presence of the NBUC with a core with a high current velocity (>0.6 m.s^-1^) below ~100 m [28]. Organisms may avoid this layer to limit horizontal advection (Fig 9A–9D). On the contrary, the oxygen concentration is likely not a driving factor since the entire water column is well oxygenated.

### 4.2. Mesopelagic layer

As in most oceanic regions, we observed more than one SSLs in the mesopelagic layer. The acoustic seascape depends on the hydrodynamic, and biological features of a given ocean region [54, 64, 65]. Here, we examine the overall relationships between the mesopelagic SSLs (composite profile) and the oxygen concentration, stratification, and current. Unfortunately, light penetration measurements were not conducted during the surveys, we therefore cannot investigate its potential effect in the distribution of SSLs.

We observed both migrant and non-migrant SSLs. This last one was primarily observed in the range ~500–650 m (Fig 9). Invertebrates such as euphausiids, salps, pyrosomes, and siphonophores abundant in the area according to our results (Tables 3 and 4; [61]), are also known to perform DVM (e.g., [66–68]). Three mesopelagic fish families dominated the area: Gonostomatidae, Myctophidae, Sternoptychidae [69–72]. Previous studies based on the same oceanographic surveys that we are used here [65–68] and others (e.g. [67]) show that these animals exhibit a variety of diel patterns ranging from species performing large vertical migrations (e.g., most hatchetfishes and lanternfishes; [65, 68]) to non-migrant species that remain full-time in mesopelagic layers, in particular at ~500–650 m (e.g., *Cyclotone spp*; [71, 72]). Additionally, they show that various fish species (e.g., lanternfishes) performing DVM present an asynchronous pattern of migration, where the entire population does not respond synchronously to diel variation [72] and only the hungry portion of the population migrates in a given day [69, 70, 72].

In the SECS (Fig 8E–8H), subsurface waters were overall less oxygenated (2 > O2 < 3.8 ml.l^-1^), including specific layers presenting the lower oxygen concentration, due to the influence of eastern waters brought by the cSEC [73]. We also observed that, except in spring 2015 at night, SSLs were negatively correlated to dissolved oxygen concentration, we found stronger SSLs at less oxygenated depths. Further, our results show that the correlation was stronger in autumn 2017 when the water column was less oxygenated. However, some fish species have been observed in the less oxygen layer during the day (e.g., *Argyropelecus affinis* and *A*. *sladani*) or all along the diel cycle (e.g., *Argyropelecus hemigymmus*) [69, 70]. Organisms may concentrate in search for cold and less oxygenated waters as a predator refuge and/or saving energy strategy [74–76]. In addition, organisms respiration may locally deplete the oxygen concentration, creating a local minimum [23]. Our results agree with several previous studies (e.g., [20, 23, 75, 77]) showing that in areas without midwater OMZs, deep SSLs are often observed in depths with less oxygen conditions in the water column, especially during the day. Otherwise, in areas with midwater OMZs, most backscatter happens at depths above the anoxic layer during the day [23, 78].

In the WBCS, the water column is well oxygenated due to the influence of the central water from the southwestern subtropical South Atlantic that is carried to the region by the NBUC [79, 80]. Therefore, oxygen concentration is likely not a potential driving factor in this system. However, in this system, the strong NBUC may impact organism distribution. The NBUC core was thicker and stronger in spring 2015 than autumn 2017 [28]. The vertical distribution of SSLs in the WBCS (Fig 5) suggests that in spring, when the NBUC is most intense, most organisms seem to avoid the core of the NBUC and distribute deeper (below 500 m) than in autumn (300–400 m) when the NBUC is weaker (Fig 8A–8D). SSLs were then associated with layers of strong stratification (more in autumn at day), indicating that this factor is likely a key potential driver not only in the thermocline layer but also below. A similar impact of stratification was observed in the SECS. In this system, an interesting pattern was observed concerning the current with a positive correlation in spring when the undercurrent, SEUC, was stronger and the opposite in autumn. This pattern is mainly related to SSLs around 300 m over the influence of the core of the undercurrent SEUC, which were also correlated to low oxygen concentrations. However, these SSLs showed slightly higher densities in the autumn than in the spring.

## 5. Conclusion

By investigating the fine-scale relationships between the vertical biophysical factors on the vertical structuring of acoustic biomass in the SWTA, we show that chlorophyll-*a*, oxygen, season (in the form of stratification and hydrodynamics) show strong correlations with the vertical distribution of SSLs, presenting themselves as potential acoustic scape drivers. However, their relative importance depends on the area, the depth range, the diel cycle and, consequently, of the seasons due to the large hydrodynamic variability observed between spring and autumn. Chlorophyll levels and depth of chlorophyll maximum may influence both in situ optical conditions as well as the vertical distribution of primary consumers. Oxygen content influences the metabolic rates of organisms. Vertical stratification of the water column and horizontal current shear can often act as obstacles to vertical movement of organisms, indirectly influencing the energy expenditure for this and also the degradation of some colonies of gelatinous organisms. However, even without a detailed mechanistic understanding, the number of correlations observed here may suggest mechanisms that will improve current models of mesopelagic organism behaviour. In the epipelagic layer, the strongest acoustic responses were associated with depths of greatest stability (highest stratification). Even in the WBCS, where the chlorophyll-*a* peaks were deeper than the peak of stratifications, SSLs were more correlated with stratification than with the deep chlorophyll-*a* maximum. Dissolved oxygen does not seem to be a key factor in the WBCS where the entire water column is well oxygenated while it appears to be a driver in the SECS where lower oxygen concentrations occur on sub-surface. Finally, our results suggest that organisms seem to avoid the core of the currents, especially the strong NBUC. However, future works are needed to better understand the role of currents on the vertical distribution of organisms and intensive ground truthing of the acoustic signal will be necessary in the future in order to reduce the uncertainties in species identification.

## Supporting information

S1 Fig Mean Composite profile acoustic profiles in spring 2015 and autumn 2017 for each diel period (day–dark colours; night–shaded colours) and system. The respective profiles are composed of the frequency (38 kHz in red, 70 kHz in green and 120 kHz in blue) providing the highest backscatter at each depth. The compositions are divided between the epipelagic zone (above 200 m) and the mesopelagic zone where only 38 and 70 kHz are available.
(TIF)

S2 Fig Example of a CTD profile from WBCS spring 2015 (ABRACOS I) with the representation of the thermohaline structure defined from temperature (*ɵ*), salinity, density (*σ*) and buoyancy frequency (N^2^). With additional chlorophyll-*a* profile, where the vertical vertical shift between maximum stratification and deep chlorophyll maximum (DCM) can be clearly observed. MLD: mixed layer depth; UTD: upper thermocline depth; LTD/LPD: lower thermocline/pycnocline depth; BL: barrier layer.
(TIF)

S3 Fig Examples of MVBS (dB re 1m^-1^) profiles at 38, 70 and 120 kHz from autumn 2017 (ABRACOS 2) in the epipelagic zone of the SECS. In addition, stratification (N^2^) and chlorophyll-*a* profiles for the same stations. In highlight are superimposed the vertical shifts between the stratification and the Composite profile. MLD: mixed layer depth; UTD: upper thermocline depth; LTD/LPD: lower thermocline/pycnocline depth.
(TIF)

S1 Table Cross-correlations and the vertical shift of maximum correlation between the stratification (N^2^) and chlorophyll (mg. m^-^^3^), and also between the MVBS (dB re 1m^-1^) profiles and the stratification, chlorophyll (mg. m^3^) and oxygen (ml.l^-1^). All analyses are presented separately for each hydrodynamic system (WBCS; SECS) in relation to seasons, diel period, depth range and frequencies (38, 70 and 120 kHz).
(XLSX)
