# Supplementary figures and images for: Fine-scale vertical relationships between environmental conditions and sound scattering layers in the Southwestern Tropical Atlantic
Source: PLoS One. 2023 Aug 4;18(8):e0284953. doi: 10.1371/journal.pone.0284953 (PMC10403096; doi:10.1371/journal.pone.0284953)

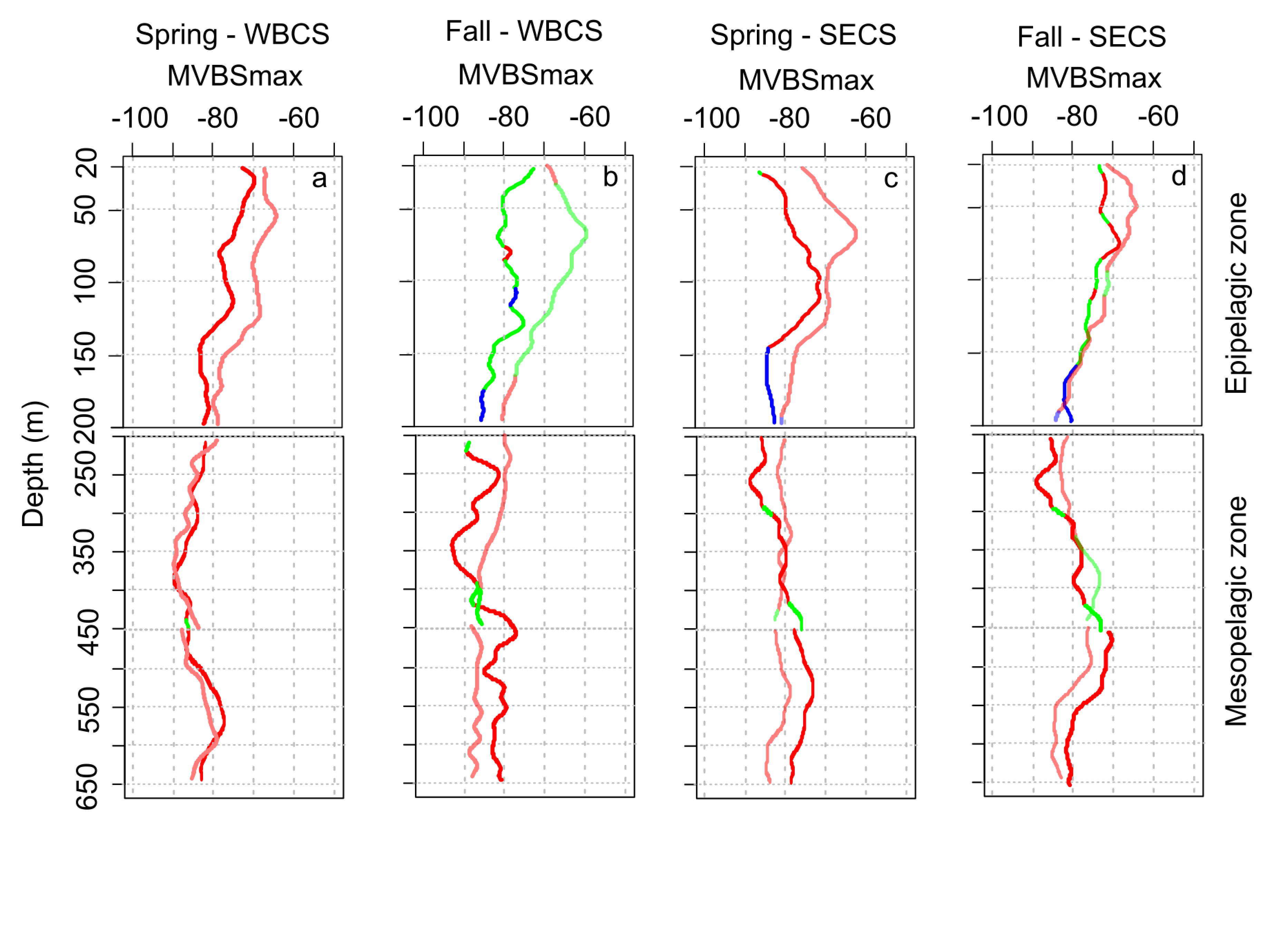

Supplement: S1 Fig — The respective profiles are composed of the frequency (38 kHz in red, 70 kHz in green and 120 kHz in blue) providing the highest backscatter at each depth. The compositions are divided between the epipelagic zone (above 200 m) and the mesopelagic zone where only 38 and 70 kHz are available. (TIF) [file pone.0284953.s001.tif]

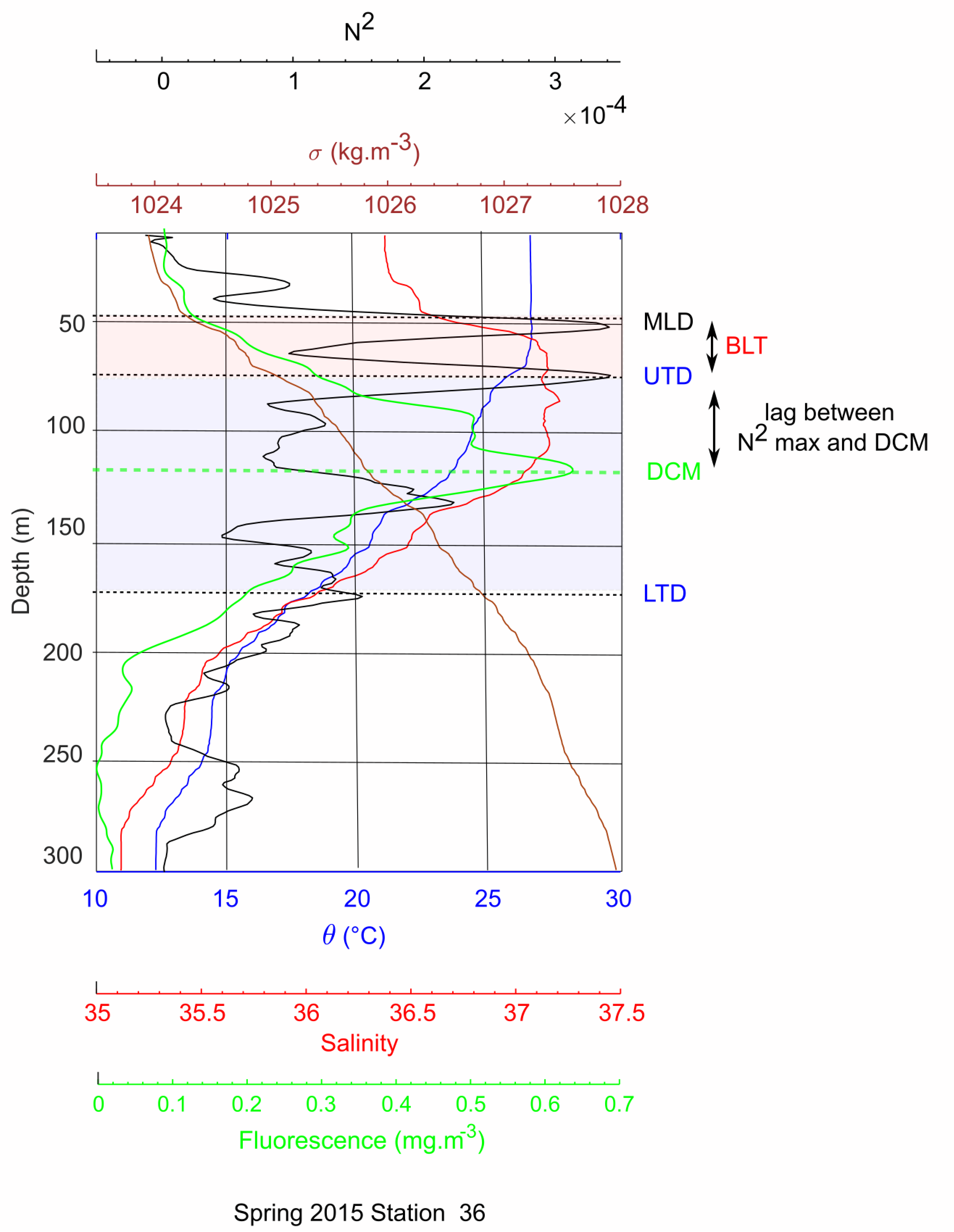

Supplement: S2 Fig — With additional chlorophyll-a profile, where the vertical vertical shift between maximum stratification and deep chlorophyll maximum (DCM) can be clearly observed. MLD: mixed layer depth; UTD: upper thermocline depth; LTD/LPD: lower thermocline/pycnocline depth; BL: barrier layer. (TIF) [file pone.0284953.s002.tif]

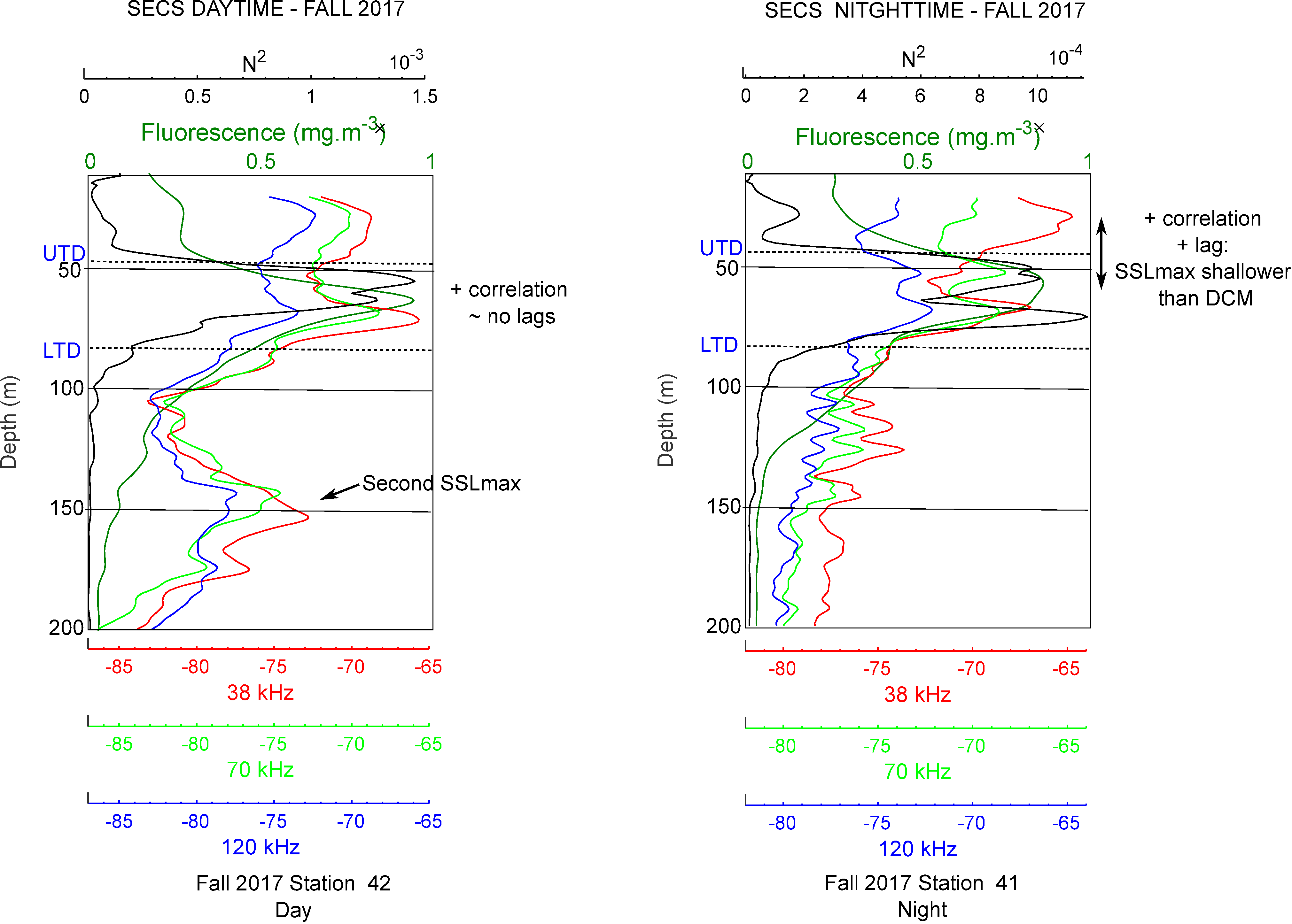

Supplement: S3 Fig — In addition, stratification (N2) and chlorophyll-a profiles for the same stations. In highlight are superimposed the vertical shifts between the stratification and the Composite profile. MLD: mixed layer depth; UTD: upper thermocline depth; LTD/LPD: lower thermocline/pycnocline depth. (TIF) [file pone.0284953.s003.tif]
